# Solid-Phase Extraction and Simultaneous Determination of Tetracycline Residues in Edible Cattle Tissues Using an HPLC-FL Method

**Published:** 2012

**Authors:** Mehran Mesgari Abbasi, Mahboob Nemati, Hossein Babaei, Masoud Ansarin, Ashraf-O-Sadat Nourdadgar

**Affiliations:** a *Drug Applied Research Center, Tabriz University of Medical Sciences, Tabriz, Iran.*; b *Faculty of Pharmacy, Tabriz University of Medical Sciences, Tabriz, Iran.*; c *Health Center, Tabriz University of Medical Sciences, Tabriz, Iran.*

**Keywords:** Tetracycline, Meat, Liver, Kidney, HPLC

## Abstract

In this assay, edible cattle tissues from local markets of Ardabil, a Province of Iran, were examined for residues of tetracycline antibiotics (tetracycline, oxytetracycline and chlortetracycline). In total, 110 samples of triceps, gluteal muscle, diaphragm, kidney and liver were randomly obtained from the local markets of the city of Ardabil. Solid-phase extraction (SPE) and high-performance liquid chromatography (HPLC) methods were used to extract and analyze tetracycline antibiotic (TC) residues, respectively.

The mean amount of total TC residues in all tested samples was 226.3 ± 112.5 ng/g and the mean amount of the total TC residues in triceps, gluteal muscle, diaphragm, kidney and liver samples were 176.3 ± 46.8, 405.3 ± 219.6, 96.8 ± 26.9, 672.4 ± 192.0 and 651.3 ± 210.1 ng/g, respectively. Additionally, 25.8% of muscle samples, 31.8% of liver samples and 22.7% of kidney samples contained amounts of TC residues beyond the maximum residue limit (MRLs). To reduce the TC residues found in edible cattle tissues, regulatory authorities should ensure that the cattle would undergo the proper withdrawal period from TCs before the slaughtering.

## Introduction

Tetracyclines are antibiotics with broad antibacterial spectrums and bacteriostatic activity against both Gram-positive and Gram-negative bacteria as well as intracellular Mycoplasma, Rickettsia and Chlamydia (1-3). These antibiotics are widely used in animal husbandry for both the prevention and treatment of diseases as well as to promote the growth. In food-producing animals, tetracyclines may be administrated orally in food or drinking water, parenterally, or through the intramammary infusion. Due to the enterohepatic circulation, tetracycline antibiotic (TC) residues may persist in the body long after the administration. The levels of TC residues in animal products depend on the initial dosage and the duration between the drug administration and animal product collection. This timeframe is called the withdrawal or washout period (1). Antibiotic residues, such as residues of other drugs, can remain in an animal’s body even after the slaughtering if the antibiotic withdrawal period is insufficient (2). Antibiotic residues in foods can influence the bacterial composition and the metabolic activity of the intestinal microflora of the consumer as well as the consumer’s metabolism of endogenous compounds (4). TC residues in meat may stain the teeth of young children (4). The presence of antibiotic residues in meat, milk and other food products may cause allergic reactions in sensitive individuals and drive the development of resistant strains of bacteria due to the ingestion of subtherapeutic doses of antibiotics (5-9). To ensure the human food safety, the World Health Organization (WHO) and the Food and Agriculture Organization (FAO) have set standards for acceptable daily intake (ADI) and maximum residue limits (MRLs) in foods. Additionally, the European Union (EU), the United States of America (USA), Canada and some other countries have set their own MRLs (2). The acceptable MRLs for TC residues (individually or in combination), as recommended by the Joint FAO/WHO Expert Committee on Food Additives, are 200, 600 and 1200 ng/g for muscles, liver and kidney, respectively (10).

Some methods, including microbiological and chromatographic methods, have been adapted for monitoring the TC residues in edible tissues and other food samples. Microbiological assays are most commonly used to analyze antibiotic residues are the. However, these assays are time-consuming, nonspecific and occasionally produce false positives (11). In contrast, chromatographic techniques, such as thin layer chromatography (TLC), capillary electrophoresis (CE) and high-performance liquid chromatography (HPLC), have been developed for the quantitative, accurate and reliable measurements of TC residues in animal tissues (11).

Oxytetracycline residue was found to be present in levels higher than those tolerated by the EU and FDA in seven of ten cured meat samples from Turkey analyzed through an HPLC method (12). In 2001, 45.6% of meat samples from Nairobi slaughterhouses had detectable TC residues and 20% of them had residue levels above the WHO standard (13). Dipleolu reported 15.6% positive TC residues of goat meat samples from two states in Nigeria (14). In a study carried out in 2006 in Hanoi, 5.5% of all meat samples were positive for TC residues and the MRLs were exceeded in 0.69% of the samples (15). Studies in Kuwait showed that none of the tested meat samples had TC residues above the acceptable limits (16). Additionally, 21.7% of all samples and 5% of kidney and liver samples from slaughterhouses in Tabriz, (Tabriz, Iran) contained TC residues above the MRLs set by the WHO (17). In addition, Ehsani and colleagues were reported the results of a study on broiler meats in Ahvaz, Iran. They showed that 60% of the samples had contamination and tetracycline residue was significantly higher than European legal concentration (100 ug/Kg) in 10% of samples (18).

The aim of this study was to investigate the presence of TC residues in various marketed edible cattle tissues in the markets of Ardabil.

## Experimental

*Chemicals and reagents*

Analytical standards of TCs, chemicals, and an Oasis HLB cartridge (WAT106202) were purchased from Sigma-Aldrich (St. Louis, USA), Merck (Darmstadt, Germany), and Waters (Milford, USA), respectively. HPLC-grade methanol and acetonitrile were purchased from Merck (Darmstadt, Germany) and double-distilled and deionized water were prepared using a Millipore water purification unit (Billerica, USA).

*Sampling procedure*

Sampling was carried out using a stratified random sampling method and 110 edible cattle tissue samples were obtained from indoor markets in different areas of Ardabil. Ultimately, 22 samples of different tissues, including triceps, diaphragm, gluteal muscle, kidney and liver were obtained. At least, 50 g of each sample was placed in a sterile polypropylene bag and kept in a -70°C deep freezer (Snijders Scientific, Holland) until analysis.

*Sample preparation*

Samples were prepared using the method reported previously by McDonald and Bouvier (19). In brief, the samples were cut into fine pieces and 5 g of each was homogenized and diluted with 20 mL of McIlvaine buffer (mixed citrate/phosphate with pH of 4.1 and EDTA). The mixtures were put in a high-intensity sonicator for 12 min followed by shaking for 10 min. The homogenized samples were centrifuged at 10,000 g for 10 min at 4°C and then re-diluted with McIlvaine buffer. The mixtures were then centrifuged again. Next, the supernatants were filtered through a 0.45 μ filter (Nalgene, USA). A solid-phase extraction method was used to extract TC residues as follows:

An Oasis HLB cartridge (WAT106202) was conditioned with 3 mL of methanol and then rinsed with 2 mL of deionized water. The filtered supernatant was loaded in the Oasis HLB cartridge at a flow rate of 5 mL/min. The cartridge was washed with 2 mL of 5% methanol in deionized water. The elution of TC residues was performed with 3 mL of HPLC grade methanol at a flow rate of 5 mL/min. Samples were lyophilized to dry and then reconstituted with 1 mL of mobile phase. Concentrations and dilution factors were considered to calculate the real amount of TC residues in samples after the analysis (13, 17- 22).

*Preparation of standard curves*

**Figure 1:**
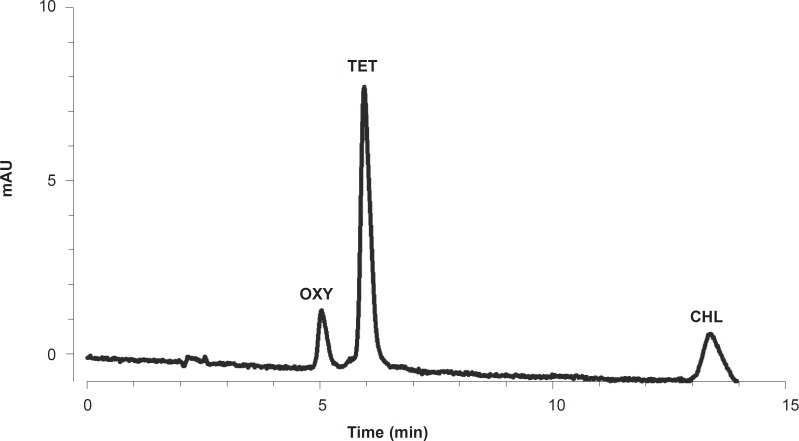
An HPLC chromatogram of a mixed standard solution of TCs (3333 ng/g

Standard solutions were prepared freshly through dissolving TCs in a mixture of methanol, acetonitrile and 50 mM oxalic acid (10:20:70, v/v). Serial dilutions were prepared for each of the TCs (oxytetracycline, tetracycline and chlortetracycline) to give 10, 50, 100, 500, 1,000, 5,000, 10,000 and 50,000 ng/g concentrations, which were used to construct the standard curves. Mixed standard solutions were prepared for simultaneous calibration and calculation of TC residues. The chromatogram of a mixed standard solution of TCs at 3,333 ng/g concentration is shown in Figure 1.

*HPLC system and procedure*

HPLC was carried out on a KNAUER system (KNAUER, Berlin, Germany). The system was equipped with a quaternary pump (K-1000), a BIOTECH model 2003 degasser, a Spark Triathlon autosampler and a RF-551 fluorescence detector. Data processing was performed using the ChromGate V3.1 software.

The mobile phase was a mixture of methanol, acetonitrile and 50 mM oxalic acid (10:20:70, v/v). The prepared mobile phase was filtered through a 0.45 μ filter (Nalgene, USA) and then degassed through sonication for 5 min before the application. Detection was carried out using 255 nm and 365 nm as the excitation and emission wavelengths, respectively. A Phenomenex Luna 5 μ C18 column (Torrance, CA, USA) was used. The flow rate was 1 mL/min with an injection volume of 20 μL (17).

**Figure 2:**
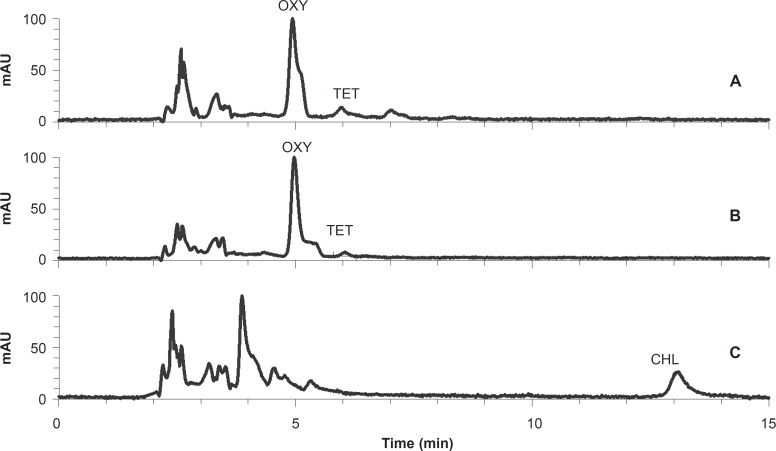
The HPLC chromatograms of a meat sample (A) and a liver sample (B) containing oxytetracycline and tetracycline residues, and a kidney sample (C) containing chlortetracycline residue.

The quantitation was based on the linear (R^2^ > 0.999) calibration curves derived from the concentrations and measured peak areas of the standard solutions. TC residue levels were extrapolated from the calibration curves. HPLC chromatograms of two tissue samples containing tetracycline and chlortetracycline residues are shown in Figure 2. The limit of detection (LOD) and the limit of quantification (LOQ) for TC residues were 2.2 and 6.6 ng/g, respectively. The recovery, inter-day and intra-day variations of the method were calculated as shown in Table 1.

**Table 1:** The recovery, intra-day, and inter-day variations of the method used for the detection of TC residues

**Parameter**	**Antibiotic**			
	**Oxytetracycline** n = 9	**Tetracycline** n = 9	**Chlortetracycline** n = 9	**Total mean** n = 27
**Mean recovery (%)**	76.8	74.8	73.0	74.9
**Intra-day variation** mean C.V. (%)	3.7	4.0	4.5	4.1
**Inter-day variation** mean C.V. (%)	4.1	4.5	5.1	4.6

##  Results and Discussion

The mean of oxytetracycline residues in all 110 samples was 157.1 ± 51.1 ng/g and the maximum amount was 4201.8 ng/g. In total, 45.5% of the samples had detectable oxytetracycline residue (Figure 2B). The mean amounts of oxytetracycline residue in triceps, diaphragm, gluteal muscle, kidney and liver samples were 86.0 ± 27.2, 73.8 ± 26.8, 302.9 ± 234.9, 187.3 ± 81.9 and 135.3 ± 52.3 ng/g, respectively. The mean value of oxytetracycline residue in all meat samples was 154.2 ± 79.2 ng/g (Table 2).

The mean amount of tetracycline residue in all analyzed samples was 13.6 ± 6.1 ng/g, with 24.5% of the samples having measurable amounts of tetracycline residue, and the maximum amount was 642.7 ng/g. The mean amounts of tetracycline residue in triceps, diaphragm, gluteal muscle, kidney and liver samples were 29.7 ± 29.1, 7.5 ± 4.2, 6.7 ± 4.1, 19.1 ± 6.1 and 5.3 ± 3.5 ng/g, respectively. The mean value of this antibiotic residue in all meat samples was 14.6 ± 9.8 ng/g (Table 2).

**Table 2:** The mean amount of TC residues in meat, liver and kidney samples

**Sample**	**Antibiotic**			
	**Oxytetracycline** **(ng/g)**	**Tetracycline** **(ng/g)**	**Chlortetracycline** **(ng/g)**	**Total TCs** **(ng/g)**
**Triceps muscle** n = 22	86.0 ± 27.2	29.7 ± 29.1	60.7 ±19.5	176.3 ± 46.8
**Diaphragm muscle** n = 22	73.8 ± 26.8	7.5 ± 4.2	15.4 ± 9.5	96.8 ± 26.9
**Gluteal muscle** n = 22	302.9 ± 234.9	6.7 ± 4.1	96.4 ± 77.6	405.3 ± 219.6
**Mean all muscles** n = 66	154.2 ± 79.2	14.6 ± 9.8	57.5 ± 26.8	226.3 ± 112.5
**Liver** n = 22	187.3 ± 81.9	19.1 ± 6.1	444.8 ± 140	651.3 ± 210.1
**Kidney** n = 22	135.3 ± 52.3	5.3 ± 3.5	531.9 ± 162.7	672.4 ± 192.0
**Total Mean** n = 110	157.1 ± 51	13.6 ± 6.1	229.8 ± 49.5	396.9 ± 77.5

Data are presented as mean **± **standard error of the mean and n indicates the number of samples

In the analyzed samples, the mean amount of chlortetracycline residue was 229.8 ± 49.5 ng/g and 40.9% of samples had detectable chlortetracycline residue. The mean amounts of chlortetracycline residue in triceps, diaphragm, gluteal muscle, kidney and liver samples were 60.7 ±19.5, 15.4 ± 9.5, 96.4 ± 77.6, 444.8 ± 140.1 and 531.9 ± 162.7 ng/g, respectively. The mean amount of this drug residue in all meat samples was 57.5 ± 26.8 ng/g (Table 2).

The mean amount of total (sum) TC residues in all samples was 396.9 ± 77.5 ng/g and the maximum amount was 4201.8 ng/g. The mean amount of total TC residues in triceps, diaphragm, gluteal muscle, kidney and liver samples were 176.3 ± 46.8, 96.8 ± 26.9, 405.3 ± 219.6, 651.3 ± 210.1 and 672.4 ± 192.0 ng/g, respectively (Table 2). The mean amount of total detected residues in the three muscle samples was 226.3 ± 112.5 ng/g and 38.2% of these muscle samples lacked detectable amounts of TC residues. Figure 2 shows representative chromatograms of a meat sample (A) and a liver sample (B) containing tetracycline and oxytetracycline residues, and a kidney sample (C) containing chlortetracycline residue.

In this study, the overall recoveries of TC residues from bovine muscle, kidney and liver ranged from 73 to 77% with the coefficient of variation ranging from 3.7 to 5.1% (n = 27) and a calculated limit of detection (LOD) of 2.2 ng/g. By comparison, previous studies reported the recovery and detection limit of TC residues in animal tissues using electrospray tandem mass spectrometry of 70-115% and 2 ng/g (21) and using an HPLC method, were reported to be 78-100%, and 2 - 10 ng/g, respectively (2, 12, 13).

Of the meat samples analyzed in this study, 9.1% of all samples, 18.2% of triceps, 13.6% of diaphragm and 18.2% of gluteal muscle had oxytetracycline residue levels above the WHO standard (200 ng/g). Additionally, 9.1% of the liver samples tested in this study had oxytetracycline residues above the WHO standard. None of the kidney samples had oxytetracycline residues above the WHO standard.

With respect to the tetracycline residue, 1.5% of all meat samples and 4.5% of triceps muscle samples had levels above the WHO standard (200 ng/g). None of the diaphragm, gluteal muscle, kidney or liver samples had tetracycline residues above the WHO standard level.

 Regarding the chlortetracycline residue, 6.1% of all meat samples, 9.1% of triceps and 9.1% of gluteal muscle samples had levels above the WHO standard (200 ng/g). Chlortetracycline residues greater than the WHO standard levels were observed in 27.3% of liver and 22.7% of kidney samples as well. However, the diaphragm samples did not have chlortetracycline residues above the WHO standard level.

With respect to TC residue, 25.8% of all meat samples, 31.8% of triceps, 13.6% of diaphragm and 27.3% of gluteal muscle samples had amounts (sum) above the WHO standard (200 ng/g). In addition, total TC residues were above the WHO standard level in 22.7% of kidney and 31.8% of liver samples, respectively (Table 3).

**Table 3:** The number and percent of samples with TC residues above the WHO MRLs

**Sample** (WHO MRLs)	**Antibiotic**			
	**Oxytetracycline** n (%)	**Tetracycline** n (%)	**Chlortetracycline** n (%)	**Total TCs** n (%)
**Triceps muscle** (> 200 ng/g)	4 (18.2)	1 (4.5)	2 (9.1)	7 (31.8)
**Diaphragm muscle** (> 200 ng/g)	3 (13.6)	-	-	3 (13.6)
**Gluteal muscle** (> 200 ng/g)	4 (18.2)	-	2 (9.1)	6 (27.3)
M**ean all muscles** (> 200 ng/g)	9 (9.1)	1 (1.5)	4 (6.1)	17 (25.8)
**Liver** (> 600 ng/g)	2 (9.1)	-	6 (27.3)	7 (31.8)
**Kidney** (> 1200 ng/g)	**-**	**-**	5 (22.7)	5 (22.7)

The mean value of total TC residues in gluteal muscle samples was greater than that of triceps samples, and the mean value in triceps samples was greater than that of diaphragm samples. These levels may correlate with the vicinity of the sampling points to the drug injection points.

The mean level of TC residues was highest in kidney samples. This difference is likely, since the major elimination pathway of tetracycline is via renal excretion, with approximately 60% of tetracycline administrated being excreted in the urine in an unchanged form (3).

## Conclusions

This study demonstrates that TC resides are present in edible cattle tissues marketed in the city of Ardabil. A considerable percentage of the tested samples contained TC residues above the MRLs. This could be a result of the common misuse of TCs in Ardabil due to the economical advantages they offer to farmers. The application of antibiotics for treatment and prevention, and as a food additive by farmers without veterinary diagnosis, prescription or supervision is frequent and must be firmly dealt with.

High levels of TC residues in edible tissues may produce drug resistance, digestive and allergic effects, and other harmful effects in consumers. These levels could also change the organoleptic properties of some meat samples. Therefore, the edible cattle tissues marketed in Ardabil are not optimal given the presence of tetracycline residues that are greater than the prescribed MRLs. Responsible authorities of the province should consider these results, be aware of the health issues they present, collaborate with the Ministry of Health and, with clear policies and procedures, undertake a thorough safety review of TC residues. More studies are necessary to evaluate other drug residues in edible tissues and to evaluate the hazard these residues present with daily intake.
